# The outcome of treatment of chronic osteomyelitis according to an integrated approach

**DOI:** 10.1007/s11751-016-0259-1

**Published:** 2016-07-01

**Authors:** Leonard C. Marais, Nando Ferreira, Colleen Aldous, Theo L. B. Le Roux

**Affiliations:** 1 Tumour, Sepsis and Reconstruction Unit, Department of Orthopaedic Surgery, School of Clinical Medicine, Grey’s Hospital, University of KwaZulu-Natal, Private Bag X9001, Pietermaritzburg, 3201 South Africa; 2 School of Clinical Medicine, College of Health Sciences, University of KwaZulu-Natal, Durban, South Africa; 3 Department of Orthopaedics, I Military Hospital, University of Pretoria, Pretoria, South Africa

**Keywords:** Osteomyelitis, Chronic, Classification, Outcome, Management

## Abstract

Previous classification systems of chronic osteomyelitis have failed to provide objective and pragmatic guidelines for selection of the appropriate treatment strategy. In this study, we assessed the short-term treatment outcome in adult patients with long-bone chronic osteomyelitis prospectively where a modified host classification system was integrated with treatment strategy selection through a novel management algorithm. Twenty-six of the 28 enrolled patients were available for follow-up at a minimum of 12 months. The median patient age of was 36.5 years (range 18–72 years). Fourteen patients (54 %) were managed palliatively, and 11 patients (42 %) were managed through the implementation of a curative treatment strategy. One patient required alternative treatment in the form of an amputation. The overall success rate was 96.2 % (95 % CI 80.4–99.9 %) at a minimum of 12-months follow-up. Remission was achieved in all [11/11] patients treated curatively (one-sided 95 % CI 73.5–100.0 %). Palliative treatment was successful in 92.9 % [13/14] of cases (95 % CI 66.1–99.9 %). In patients with lower limb involvement, there was a statistically significant improvement of 28.3 (95 % CI 21.0–35.7; SD 17.0) in the AAOS Lower Limb Outcomes Instrument score (*p* value < 0.001). The integrated approach proposed in this study appears a useful guideline to the management of chronic osteomyelitis of long bones in adult patients in the developing world. Further investigation is required to validate the approach, and additional development of the algorithm may be required in order to render it useful in other clinical environments.

## Introduction

Long-bone chronic osteomyelitis is challenging to treat in adult patients. The typical causative organisms possess characteristics that render greater resistance to the host’s immune response and antibiotic therapy. Bacteria may persist in a biofilm-based colony or be intracellular, concealed within osteoblasts [1, 2]. While chronic haematogenous osteomyelitis is not associated with skeletal instability, it frequently involves a large segment of bone. In contrast, post-traumatic contiguous osteomyelitis is complicated often by the presence of instability or a compromised soft tissue envelope. Lastly, there are systemic risk factors present in the host that compromise the ability of the immune system to combat infection effectively.

Several classification systems have been proposed, but none has been accepted universally [3, 4]. Although the Cierny and Mader classification has been the most popular, the stratification of the physiological status of the host remains problematic [5, 6]. The definition of a C-host, according to this classification, is subjective in nature and is dependent on the treating surgeon’s ability to predict the patient’s response to a therapeutic intervention [7]. The differentiation between a type B- and C-host is important as it identifies patients who should be treated curatively or palliatively [3]. In addition, the lack of standardization in host classification has made comparison with results from different studies challenging [8].

There is no evidence-based guidance on the treatment of chronic osteomyelitis in adults [3]. There is no single-treatment regimen or surgical procedure that is appropriate for all patients [9]. Essentially, the choice is between a curative, a palliative or an alternative approach. Curative treatment usually involves surgical debridement with or without complex reconstructive procedures and short-term pathogen-directed antimicrobial therapy [10]. Palliative treatment on the other hand typically involves long-term chronic suppressive antibiotic therapy (CSAT) and rarely intralesional or minimally invasive surgical intervention [11]. An alternative treatment strategy is indicated occasionally and may comprise either of amputation of the limb or a combination of surgical intervention and chronic suppressive antibiotic therapy. The main difficulty lies in choosing the correct treatment strategy for each patient, a process further complicated by the aforementioned lack of standardization in host stratification.

The limitations of existing classification systems, as well as the lack of evidence-based guidelines, prompted us to develop a classification system and treatment algorithm that would assist in treatment strategy selection in a developing country. In this study, we investigate the short-term outcome of treatment in adult patients with long-bone chronic osteomyelitis where a modified host classification system was integrated, via a novel management algorithm, with treatment strategy selection.

## Materials and methods

A prospective study was performed on 28 consecutive patients with long-bone chronic osteomyelitis treated at a tertiary-level tumour, sepsis, and reconstruction unit. All adult patients older than 18 years of age and with a minimum follow-up of 12 months were included in the series. Patients with infections involving the foot or hand, atypical organisms (including tuberculosis and fungal infections), arthroplasty-related periprosthetic infection, or early (within 90 days) post-operative surgical site infection with stable implants were excluded from the study. Data were collected with regard to patient demographics, the cause and site of infection, the initial and final impairment, causative organisms, management strategy employed, follow-up period, and outcome of treatment in terms of remission or suppression of infection. Impairment was assessed by means of the QuickDASH scoring system for upper limbs or AAOS Lower Limb Outcomes Instrument (version 2.0) in the case of lower limb involvement [12, 13].

For the purposes of this study, chronic osteomyelitis was defined as an infection involving bone, with a duration of at least 10 days, where the causative organisms were thought to have persisted either intracellularly or in interactive biofilm-based colonies. Periprosthetic infections were excluded from the study based on the current trend of classifying and treating arthroplasty-related infections as a separate entity [14]. Following clinical, radiological, and biochemical evaluation, patients were classified according to a modified version of the original Cierny and Mader classification system (Table 1) [7]. In terms of the physiological status of the host, the Cierny and Mader classification system was modified in order to provide a more pragmatic and objective definition of a C-host. A patient was classified as a C-host if one major or more than two minor risk factors were present (Table 2). In order to remove any ambiguity during classification of the anatomical nature of the disease, this was performed prior to, rather than following, the debridement. The impairment resulting from the disease and the nidus of infection was added to the classification as these factors were to be considered during the treatment selection process.

**Table 1:** Modified version of the original Cierny and Mader classification system that served to guide treatment strategy selection

Classification	Characteristic
Physiological	
Type A-host	No risk factors
Type B-host	Less than three minor risk factors
Type C-host	One major and/or three or more minor risk factors
Pathoanatomy	
I—Medullary	No cortical sequestration
II—Cortical	Direct contiguous involvement in cortex only
III—Combined (stable)	Both cortex and medullary regions involved
IV—Combined (unstable)	As for III plus unstable prior to debridement
Nidus	
Sequestrum	Cortical sequestrum present
Implant	Biofilm-based infection in the presence of implant
No identifiable nidus	Minimal necrosis osteomyelitis
Impairment	
Minimal	Patient able to perform ADL (activities of daily living)
Severe	Unable to perform ADL

**Table 2:** Risk factors used to stratify the physiological status of the host

Major risk factors	Minor systemic risk factors	Minor local risk factors
CD_4_ count <350 cells/mm^3^	HIV infection	Poor soft tissues requiring flap
Albumin <30 g/l	Anaemia	Chronic venous insufficiency
HbA1C ≥8 %	Smoking	Peripheral vascular disease
Cellulitis or abscess formation	Diabetes mellitus	Previous radiation therapy
Malignancy at site of infection	Rheumatoid arthritis	Surgery will result in instability
Pathological fracture	Chronic lung disease	Adjacent joint stiff/arthritic
	Chronic cardiac failure	Heterotopic ossification
	Paraplegia/quadriplegia	Failed reconstruction elsewhere
	Drug or substance abuse	Foot involvement
	Chronic corticosteroid use	Pelvic involvement
	Active tuberculosis	Adjacent joint involved
	Ischaemic heart disease	Segmental resection of ≥6 cm
	Cerebrovascular disease	Required to achieve cure
	Compliance and motivation	
	Age > 65 Common variable immune deficiency	

**Fig. 1:**
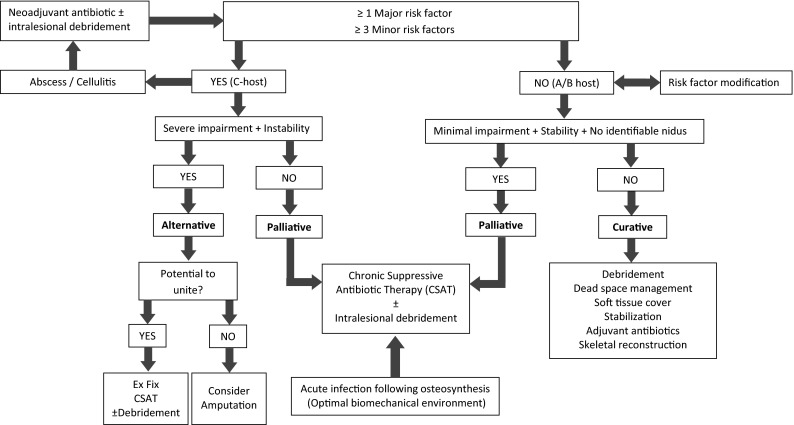
Treatment selection algorithm

The modified classification system was integrated with treatment strategy selection through the implementation of a novel management algorithm (Fig. 1). All C-hosts, as well as A- or B-hosts with minimal impairment, no identifiable source and no skeletal instability, were managed palliatively. All remaining A- and B-hosts were treated curatively. Those C-hosts with severe impairment combined with skeletal instability were managed through the implementation of an alternative treatment strategy. This involved either amputation or chronic suppressive antibiotic therapy in combination with external fixation with or without intralesional debridement.

Curative treatment involved marginal or wide resection, dead space management, provision of bony stability, soft tissue reconstruction, and/or skeletal reconstruction, in conjunction with pathogen-directed adjuvant antibiotics for a period of 6 weeks. In cases without skeletal instability (Cierny and Mader anatomical type I, II and III lesions), the aim was to maintain stability by marginal debridement through direct unroofing (tangential excision with high-speed burr) and/or indirect unroofing (medullary reaming). In cases involving skeletal instability, wide (segmental) resection was performed and stability provided by circular external fixation. Dead space management techniques were tailored to the anatomical nature of the pathology. A modified version of continuous irrigation, as proposed by Lautenbach, was used in type I (medullary) post-operative infections [15, 16]. A solution of 80 mg of gentamicin in 1000 ml 0.9 % NaCl was infused at 125 ml/h through a single perforated 6-mm drain tube that was placed intramedullary through the nail entry site and a distal cortical window at the site of the previous locking screws. The irrigation was discontinued, and drain was removed once the effluent fluid was macroscopically clear. In type III lesions (stable combined medullary and cortical lesions), gentamicin-impregnated polymethylmethacrylate (PMMA) beads (Septopal^®^ Merck, Darmstadt Germany) were used and removed at 6–8 weeks. Emphasis was placed on soft tissue reconstruction with the closure of soft tissue defects with well-perfused healthy tissue. Where direct primary closure was deemed unfeasible, a plastic surgeon performed closure with a tissue flap with preference given to muscular flaps. Post-operatively, all patients were treated with generic parenteral antibiotics in the form of cefazolin and imipenem until the 7-day microscopy, culture, and sensitivity (MCS) results became available. Oral antibiotic therapy, in the form of two agents that were tailored to the culture and sensitivity results, was commenced subsequently and continued for a period of 6 weeks.

**Fig. 2:**
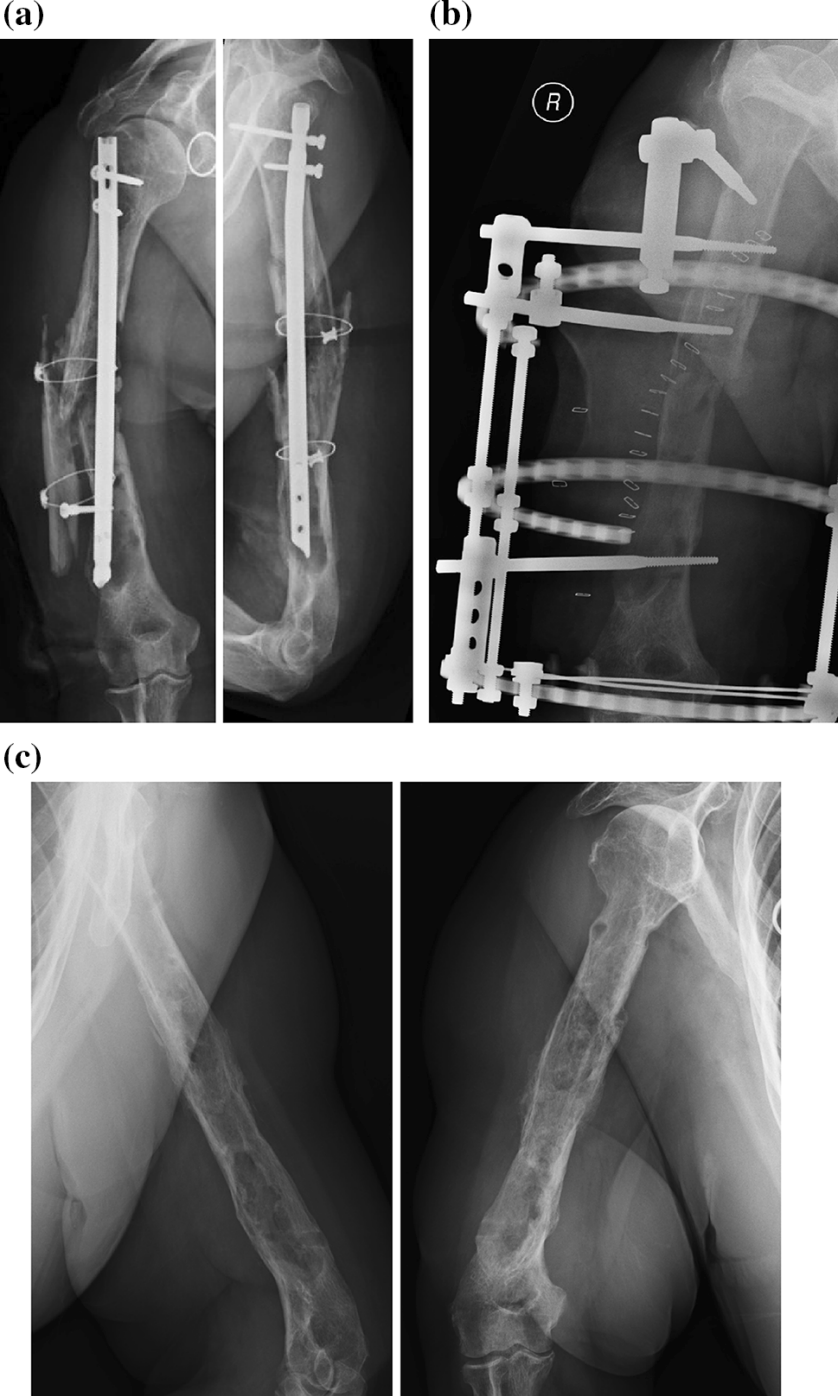
X-ray images of a case involving pre-operative instability (anatomical type IV infection). **a** This 72-year-old diabetic patient presented with a septic non-union of the humerus following multiple previous surgeries. **b** Reconstruction of the post-debridement defect involved acute shortening, bone graft, and circular external fixation. **c** Radiological images following removal of external fixator

Following this period, reconstruction of segmental bone defects in Cierny and Mader type IV lesions was undertaken if clinical and biochemical evaluation confirmed the absence of active infection. The treatment protocol dictated that the size of the bone defect would determine the nature of the subsequent skeletal reconstruction procedure. Defects less than 1–2 cm in magnitude were managed by acute shortening (Fig. 2). In long bones other than the tibia, defects between 2 and 4 cm in size were managed using the Masquelet technique, involving autogenous bone grafting into an induced membrane. Tibial defects larger than 2 cm and gaps in other long bones in excess of 4 cm were treated through the use of bone transport.

Palliative treatment involved the use of chronic suppressive antibiotic therapy (CSAT) in the form of trimethoprim–sulfamethoxazole (800 mg/160 mg twice daily) and rifampicin (600 mg daily). In cases where the general condition of the patient and local soft tissues allowed, an intralesional excision of discreet exposed sequestra was performed. In this series, all cases treated by an alternative treatment strategy required amputation of the limb.

Following a minimum 12-month follow-up period treatment, success or failure was determined. Success was defined as achievement of remission through a curative treatment strategy or attainment of suppression in patients treated palliatively. Remission was defined as the absence of clinical signs of infection [8]. Suppression was defined as subjective resolution of infection symptoms and signs from the patient’s point of view to the extent that the patient required no additional treatment. Treatment failure was defined as the failure to achieve the predetermined goal (remission or suppression). The outcome was also reported as failure if unplanned re-operation was required or if the patient was dissatisfied with the outcome.

Data were analysed using Stata 13.0 (StataCorp. 2013. Stata Statistical Software: Release 13. College Station, TX: StataCorp LP). Continuous variables were summarized using mean and standard deviation values. If the variable was skewed or outlier values were present, then the median and interquartile range were used. Categorical variables were summarized using frequency tables. Ninety-five per cent confidence intervals were constructed around sample point estimates. Changes in functional outcome score from initial assessment to final assessment were compared using a paired *t* test. A *p* value of <0.05 was considered statistically significant for all tests.

Ethical approval was obtained from the relevant ethics review boards prior to commencement of the study.

## Results

Twenty-six of the 28 enrolled patients were available for follow-up at 12 months. One patient was excluded on the basis that he was diagnosed with a surgical site infection in association with stable fixation of a tibial plafond fracture in the early post-operative period. This infection was therefore not treated as chronic osteomyelitis. The second patient excluded was a 77-year-old male with post-operative chronic osteomyelitis following cephalomedullary nailing of a subtrochanteric fracture. The patient was lost to follow-up after the initial visit, and attempts to contact the patient were unsuccessful. The median age of the remaining patients was 36.5 years (range 18–72 years; interquartile range 24 years). Seven patients had chronic haematogenous osteomyelitis, eight had post-operative infections, nine developed chronic osteomyelitis after open fractures, and two patients developed contiguous chronic osteomyelitis as a result of direct local extension. The tibial diaphysis was the most commonly involved site (Table 3). Culture results, from tissue samples taken at the time of debridement in patients who were treated curatively, revealed a variety of causative organisms (Table 4).

**Table 3:** Site of infection

Site of infection	Number of patients
Tibia diaphysis	12 (46 %)
Femur diaphysis	8 (30 %)
Tibial plateau	2 (8 %)
Tibial plafond	1 (4 %)
Humerus diaphysis	2 (8 %)
Ulna shaft	1 (4 %)

**Table 4:** Micro-organism cultured from tissue samples taken during debridement in patients treated curatively

Micro-organisms	Number of patients
*Staphylococcus aureus*	3
*Staphylococcus epidermidis*	1
*Enterobacter* sp.	1
*Streptococcus infantarius*	1
*Pseudomonas aeruginosa*	1
*Aeromonas hydrophila*	1
*Serratia* sp.	1
*Proteus mirabilis*	1
*Pantoea* sp.	1
No growth	1
Multiple organisms	1

### Classification

**Fig. 3:**
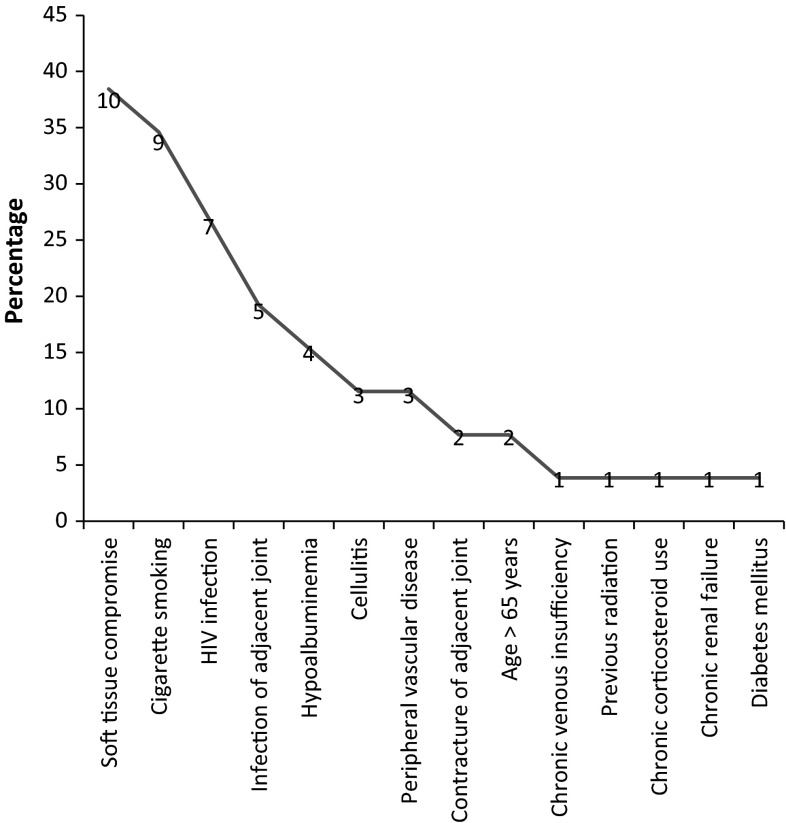
Risk factors identified in the series of cases

Three patients (12 %) were classified as A-hosts, 11 patients (42 %) as B-hosts, and 12 (46 %) as C-hosts. Six patients classified as C-hosts had at least one major risk factor and six other patients on the basis of three or more minor risk factors. Of the 12 C-hosts, six had both a major and more than two minor risk factors present. Seven patients (27 %) were HIV-positive with a mean CD_4_ count of 401 cells/mm^3^ [range 220–986 cells/mm^3^; standard deviation (SD) 238 cells/mm^3^]. A variety of additional risk factors were identified amongst the patients enrolled (Fig. 3). Nine patients (35 %) were smokers, and three patients (12 %) had hypoalbuminemia. The soft tissues in ten patients were considered to represent a significant risk factor for the development of complications following surgery unless addressed by flap or other means. Cellulitis and abscess formation, precluding definitive surgery as the first line of treatment, were present in three patients. Peripheral vascular disease or chronic venous insufficiency with lipodermatosclerosis was present in three patients. The infection involved the adjacent joint in five cases, and there was significant loss in range of motion of the adjacent joint in the additional two patients. Other risk factors included previous radiation, chronic renal failure requiring dialysis and chronic corticosteroid use in one patient, diabetes mellitus in one patient, and age over 65 years in two patients. In terms of the anatomical extent of the disease, 20 patients had type III infection, five patients had pre-operative instability, and in one patient, the infection was confined to the medullary cavity. The mean initial AAOS Lower Limb Outcomes score in patients with lower limb involvement was 58.2 (range 21–100; SD 22.9). In three cases, the upper limb was involved, with a mean initial QuickDASH score of 18.2 (range 2.3–29.5; Table 5).

**Table 5:** Functional outcome

Category	n	Mean	SD^c^	Range	*p* value^d^
Overall lower extremity^a^	23				
Initial		52	21.2	21–100	
Final		89	11.6	51–100	
Improvement		27	18.4	0–49	<0.001
Overall upper extremity^b^	3				
Initial		75	7.4	72.5–86.4	
Final		18.2	13.6	2.3–29.5	
Improvement		54.3	20.2	45.5–84.1	0.03
Palliative group^a^	14				
Initial		51.1	22.9	28–100	
Final		92.5	16.8	51–100	
Improvement		25.5	17.1	0–54	<0.001
Curative group^a^	8				
Initial		61	21.3	34–94	
Final		91	9.1	74–100	
Improvement		27.5	17.4	6–48	<0.01

^a^AAOS Lower Limb Outcomes Instrument

^b^QuickDASH

^c^Standard deviation

^d^Paired *t* test

### Management

Fourteen patients (54 %) were managed palliatively, and 11 patients (42 %) were managed through the implementation of a curative treatment strategy. One patient required alternative treatment in the form of an amputation. This patient had infection and bone loss following a neglected open fracture and was classified as a C-host on the basis of the presence of two major and two minor risk factors. The palliative treatment group comprised of 11 C-hosts and three B-hosts who had stable lesions with minimal impairment and no identifiable sequestra. All patients in the palliative treatment group received chronic suppressive antibiotic therapy—trimethoprim–sulfamethoxazole (800 mg/160 mg twice daily) and rifampicin (600 mg daily)—for a period of 3–6 months. One patient, who had an exposed sequestrum in the region of the tibial plateau, required an additional intralesional excision (simple sequestrectomy).

In the curative treatment group, surgical intervention involved marginal debridement (direct and/or indirect unroofing) in ten patients. Wide (segmental) resection of the ulna diaphysis, without subsequent reconstruction, was performed in one patient. Dead space management involved a modified Lautenbach continuous irrigation system in six cases, PMMA beads in four patients, and local muscle flap in one case. Primary soft tissue closure was obtained for all cases in the curative group. Direct primary closure of the wound was performed in ten cases, and in one instance, a local muscle flap was required. In the two patients, in whom skeletal stabilization and reconstruction were required, acute shortening and Ilizarov circular external fixation were performed. Union was achieved in both of these cases. All patients treated curatively received a combination of two oral antibiotics for a period of 6 weeks.

### Outcome

The overall success rate was 96.2 % (95 % CI 80.4–99.9 %) after a minimum of 12 months of follow-up. Remission was achieved in all [11/11] patients treated curatively (one-sided 95 % CI 73.5–100.0 %). Palliative treatment was successful in 92.8 % of cases (95 % CI 66.1–99.9 %), with suppression in 46 % and remission in the remaining 54 % of these patients. The overall mean final AAOS Lower Limb Outcomes score was 86.6 (range 51–100; SD 14.5). This equated to a statistically significant (*p* value < 0.001) mean improvement of 28.3 (95 % CI 21.0–35.7, SD 17.0). In the upper limb, the mean final overall QuickDASH score was 75 (range 72.5–86.4), with a mean improvement of 54.3 (range 45.5–84.1). There was comparable improvement in the functional outcome scores in the palliative and curative treatment groups (Table 5).

One treatment failure occurred in the palliative treatment group in a patient who required regular dialysis as a result of Goodpasture syndrome. This patient had extensive involvement in the entire femoral diaphysis after irradiation for a sarcoma, peripheral vascular disease and avascular necrosis of the femoral head. A hip disarticulation was required when the palliative treatment protocol was abandoned.

## Discussion

Chronic osteomyelitis management continues to pose a major challenge to orthopaedic surgeons [11]. The Mayo Clinic reported a 20 % failure rate in the management of chronic infections [17]. Twenty years later, the disease remains difficult to cure as was acknowledged in a recent Cochrane review on antibiotic therapy in chronic osteomyelitis [18]. The combined remission rate, in this analysis of four randomized controlled trials, was 78.8 % at 12 months. Specialized units have, however, been able to achieve superior results. Cierny, for example, achieved success in 84 % of patients managed curatively at 2-year follow-up [10]. The Bone Infection Unit in the UK reported an impressive cure rate of 90 % at 5-year follow-up [9]. While the multidisciplinary nature of the service offered by these specialized units is bound to improve outcomes, appropriate surgical candidate selection may also play a role.

Without a pragmatic and objective definition of a C-host (who should be palliated), the selection of a curative (surgical) treatment strategy, according to the Cierny and Mader classification system, is based on prior clinical experience [7]. By this approach, the expected outcome of a curative strategy should offer a distinct advantage over symptomatic treatment or amputation, in order to justify the potential morbidity and risks involved in limb salvage surgery [7, 10]. Selecting candidates for surgery on this basis requires considerable experience as it is based on a prediction of the patient’s response to treatment. The experience gained in specialized units will therefore improve the success of curative treatment strategies due to, amongst other factors, improved surgical candidate selection. The approach followed in our study was developed to serve as a guideline for treatment of chronic osteomyelitis in a resource-poor clinical environment where treatment by specialized units is not always easily accessible.

In a previous retrospective series of 109 cases, we were able to achieve an overall success rate of 90 % at a mean 18-months follow-up through an approach which integrated the pragmatic host stratification with treatment strategy selection [19]. In this study, we aimed for a preliminary validation of a similar approach prospectively. After a minimum of 12-month follow-up, we achieved an overall success rate of 96.2 %, with 100 % remission in the curative group and 92.8 % suppression (or better) in the palliative group. These results are comparable to those achieved in our retrospective series, where curative and palliative treatments were successful in 93 and 87 %, respectively [19].

Although these results appear promising, caution is advised against widespread implementation of this approach. The proposed classification system and treatment algorithm were designed for use in the developing world. It is unlikely to be suitable in the developed world without further improvement or modification. Apart from the high incidence of HIV infection and hypoalbuminemia in our series, the pattern of causative organisms identified in our cases appears to differ somewhat from that seen in the developed world [20].

Additional problems may arise when the algorithm is tested on a wider range of patients. In one case in this series, the treatment algorithm was deemed to be inadequate as it prescribed chronic suppressive antibiotic therapy (CSAT) in a C-host (on the basis of the presence of skeletal stability), where amputation was inevitable. This algorithm error was, however, on the conservative side; in many C-hosts without skeletal instability, CSAT may suppress the disease to the extent that amputation may not be required. Furthermore, the proposed host stratification criteria could result in the initiation of palliative care in patients who may have been able to cope with curative treatment. This approach may hold some benefit as it emphasizes the importance of host factor modification prior to surgical intervention. Many high-risk cases who may initially be classified as C-hosts will become candidates for curative treatment (B-hosts) following implementation of the appropriate interventions aimed at risk factor reduction.

There are further limitations to this study. The heterogeneous nature of the disease demands a much larger series of cases to determine whether the algorithm is appropriate. The follow-up period in this series is too short to determine the ultimate success rate, and our results are likely to deteriorate over time due to relapse. While deterioration can be expected in both groups, it is bound to be more pronounced in the palliative group. Long-term follow-up will be required to shed more light on this subject. The lack of a control group is a further limitation. Randomizing high-risk patients to high- or low-risk interventions, in order to identify which factors are associated failure (amputation), presents obvious ethical concerns. Future comparative studies will, however, be facilitated by the fact that we have provided a standardized host stratification system.

Despite these limitations, preliminary results suggest that our proposed approach may be useful in certain clinical environments. Our modified classification system may be more relevant to clinicians inexperienced in the management of chronic osteomyelitis as it is less dependent on estimation of the response to treatment or the prediction of instability following debridement. Another important potential benefit of this approach is that standardized host stratification may enable the comparison of results from future studies. It may become possible to compare the outcome of different interventions or strategies if the physiological host status was classified using the same pre-defined pragmatic criteria. This may, in turn, allow us to answer many of the questions that remain regarding the management of adult chronic osteomyelitis [8].

## Conclusion

The integrated approach proposed in this study appears to hold promise in the management of chronic long-bone osteomyelitis in adult patients in the developing world. Further investigation is required to validate the approach, and additional algorithm development may be required in order to render it useful in other clinical settings.
